# Supplementary material for: Tunable even- and odd-denominator fractional quantum Hall states in trilayer graphene
Source: Nat Commun. 2024 Jul 24;15:6236. doi: 10.1038/s41467-024-50589-2 (PMC11266615; doi:10.1038/s41467-024-50589-2)
Supplement: Supplementary file 1 — Supplementary information [file 41467_2024_50589_MOESM1_ESM.pdf]

Supplementary Information for

Tunable even- and odd-denominator fractional quantum Hall states in trilayer graphene

Yiwei Chen *et al.*

This Supplementary Information provides more details about the theoretical analysis of fractional quantum Hall states in trilayer graphene.

Supplementary Note 1: Models

To begin with, we define the single-particle tight-binding model for trilayer graphene (TLG). There are two sublattice sites A and B in each layer of TLG and their layer indices are appended as subscripts $1, 2, 3$. The distance between Carbon atoms is $a = 0.142$ nm and the lattice constant is $\widetilde{a} = \sqrt{3}a$. As illustrated in Fig. 1 of the main text, six hopping constants γ_i $(i = 0, 1, \cdots, 5)$ are considered in the Slonczewski-Weiss-McClure (SWMc) parametrization. The distance between layers along the vertical direction is $d = 0.335$ nm. An additional parameter δ is used to account for the onsite potential of B_1, A_2, and B_3 lattice sites. If a displacement field is present, the potential difference between the top and bottom layer is quantified by Δ_1. Based on symmetry consideration, it was proposed that another parameter Δ_2 should be introduced to describe the deviation of potential on the middle layer from the mean of the potentials on the top and bottom layers. In principle, Δ_2 could be nonzero even when Δ_1 is zero. Let us choose the basis to be $A_1, B_1, A_2, B_2, A_3, B_3$. The tight-binding Hamiltonian is

$$\mathcal{H} = \begin{bmatrix} \Delta_1+\Delta_2 & \gamma_0 t^*(\mathbf{k}) & \gamma_4 t^*(\mathbf{k}) & \gamma_3 t(\mathbf{k}) & \gamma_2/2 & 0 \\ \gamma_0 t(\mathbf{k}) & \delta+\Delta_1+\Delta_2 & \gamma_1 & \gamma_4 t^*(\mathbf{k}) & 0 & \gamma_5/2 \\ \gamma_4 t(\mathbf{k}) & \gamma_1 & \delta-2\Delta_2 & \gamma_0 t^*(\mathbf{k}) & \gamma_4 t(\mathbf{k}) & \gamma_1 \\ \gamma_3 t^*(\mathbf{k}) & \gamma_4 t(\mathbf{k}) & \gamma_0 t(\mathbf{k}) & -2\Delta_2 & \gamma_3 t^*(\mathbf{k}) & \gamma_4 t(\mathbf{k}) \\ \gamma_2/2 & 0 & \gamma_4 t^*(\mathbf{k}) & \gamma_3 t(\mathbf{k}) & -\Delta_1+\Delta_2 & \gamma_0 t^*(\mathbf{k}) \\ 0 & \gamma_5/2 & \gamma_1 & \gamma_4 t^*(\mathbf{k}) & \gamma_0 t(\mathbf{k}) & \delta-\Delta_1+\Delta_2 \end{bmatrix}, \tag{S1}$$

where $t(\mathbf{k}) = -1 - 2\cos(k_x\widetilde{a}/2)\exp(i\sqrt{3}k_y\widetilde{a}/2)$ is a summation over nearest neighbors. We adopt the parameters $\gamma_0 = 3.1$, $\gamma_1 = 0.39$, $\gamma_2 = -0.028$, $\gamma_3 = 0.315$, $\gamma_4 = 0.041$, $\gamma_5 = 0.05$, and $\delta = 0.046$ (all in units of eV) in our calculations [1]. The band structure with $\Delta_1 = \Delta_2 = 0$ is presented in Fig. 1 of the main text.

Next we turn to the Landau levels (LLs) of TLG. The energy bands have two valleys in the hexagonal Brillouin zone with momentum $\mathbf{K}_\pm = (\pm 4\pi/3, 0)$. In the vicinity of these valleys, the Hamiltonian can be expanded to yield

$$\mathcal{H}_{\mathbf{K}_+} = \begin{bmatrix} \Delta_1+\Delta_2 & v_0\pi^- & v_4\pi^- & v_3\pi^+ & \gamma_2/2 & 0 \\ v_0\pi^+ & \delta+\Delta_1+\Delta_2 & \gamma_1 & v_4\pi^- & 0 & \gamma_5/2 \\ v_4\pi^+ & \gamma_1 & \delta-2\Delta_2 & v_0\pi^- & v_4\pi^+ & \gamma_1 \\ v_3\pi^- & v_4\pi^+ & v_0\pi^+ & -2\Delta_2 & v_3\pi^- & v_4\pi^+ \\ \gamma_2/2 & 0 & v_4\pi^- & v_3\pi^+ & -\Delta_1+\Delta_2 & v_0\pi^- \\ 0 & \gamma_5/2 & \gamma_1 & v_4\pi^- & v_0\pi^+ & \delta-\Delta_1+\Delta_2 \end{bmatrix} \tag{S2}$$

and

$$\mathcal{H}_{\mathbf{K}_-} = \begin{bmatrix} \Delta_1+\Delta_2 & -v_0\pi^+ & -v_4\pi^+ & -v_3\pi^- & \gamma_2/2 & 0 \\ -v_0\pi^- & \delta+\Delta_1+\Delta_2 & \gamma_1 & -v_4\pi^+ & 0 & \gamma_5/2 \\ -v_4\pi^- & \gamma_1 & \delta-2\Delta_2 & -v_0\pi^+ & -v_4\pi^- & \gamma_1 \\ -v_3\pi^+ & -v_4\pi^- & -v_0\pi^- & -2\Delta_2 & -v_3\pi^+ & -v_4\pi^- \\ \gamma_2/2 & 0 & -v_4\pi^+ & -v_3\pi^- & -\Delta_1+\Delta_2 & -v_0\pi^+ \\ 0 & \gamma_5/2 & \gamma_1 & -v_4\pi^+ & -v_0\pi^- & \delta-\Delta_1+\Delta_2 \end{bmatrix} \tag{S3}$$

where $\hbar v_i = 3a\gamma_i/2$, $\pi^- = \hbar(k_x - ik_y)$, and $\pi^+ = \hbar(k_x + ik_y)$. For our purpose, it is convenient to change the basis to

$$\frac{A_1 - A_3}{\sqrt{2}}, \frac{B_1 - B_3}{\sqrt{2}}, \frac{A_1 + A_3}{\sqrt{2}}, \frac{B_1 + B_3}{\sqrt{2}}, A_2, B_2 \tag{S4}$$

such that $\mathcal{H}_{\mathbf{K}_\pm}$ has the block form

$$\begin{bmatrix} \mathcal{H}_{\text{MLG}} & \mathcal{H}_{\text{mix}} \\ \mathcal{H}_{\text{mix}}^\dagger & \mathcal{H}_{\text{BLG}} \end{bmatrix} \tag{S5}$$

with a two-dimensional monolayer graphene (MLG) part $\mathcal{H}_{\text{MLG}}$, a four-dimensional bilayer graphene (BLG) part $\mathcal{H}_{\text{BLG}}$, and a mixing part

$$\mathcal{H}_{\text{mix}} = \begin{bmatrix} \Delta_1 & 0 & 0 & 0 \\ 0 & \Delta_1 & 0 & 0 \end{bmatrix}. \tag{S6}$$

This representation clearly shows that the TLG Hamiltonian can be decomposed to a combination of MLG and BLG parts in the absence of Δ_1. In the $\mathbf{K}_+$ valley, we have

$$\mathcal{H}_{\text{MLG}}^+ = \begin{bmatrix} -\frac{\gamma_2}{2} + \Delta_2 & v_0\pi^- \\ v_0\pi^+ & -\frac{\gamma_5}{2} + \delta + \Delta_2 \end{bmatrix}, \qquad \mathcal{H}_{\text{BLG}}^+ = \begin{bmatrix} \frac{\gamma_2}{2} + \Delta_2 & v_0\pi^- & -\sqrt{2}v_4\pi^- & \sqrt{2}v_3\pi^+ \\ v_0\pi^+ & \frac{\gamma_5}{2} + \delta + \Delta_2 & \sqrt{2}\gamma_1 & -\sqrt{2}v_4\pi^- \\ -\sqrt{2}v_4\pi^+ & \sqrt{2}\gamma_1 & \delta - 2\Delta_2 & v_0\pi^- \\ \sqrt{2}v_3\pi^- & -\sqrt{2}v_4\pi^+ & v_0\pi^+ & -2\Delta_2 \end{bmatrix}. \tag{S7}$$

In the $\mathbf{K}_-$ valley, we have

$$\mathcal{H}_{\text{MLG}}^- = \begin{bmatrix} -\frac{\gamma_2}{2} + \Delta_2 & -v_0\pi^+ \\ -v_0\pi^- & -\frac{\gamma_5}{2} + \delta + \Delta_2 \end{bmatrix}, \qquad \mathcal{H}_{\text{BLG}}^- = \begin{bmatrix} \frac{\gamma_2}{2} + \Delta_2 & -v_0\pi^+ & \sqrt{2}v_4\pi^+ & -\sqrt{2}v_3\pi^- \\ -v_0\pi^- & \frac{\gamma_5}{2} + \delta + \Delta_2 & \sqrt{2}\gamma_1 & \sqrt{2}v_4\pi^+ \\ \sqrt{2}v_4\pi^- & \sqrt{2}\gamma_1 & \delta - 2\Delta_2 & -v_0\pi^+ \\ -\sqrt{2}v_3\pi^+ & \sqrt{2}v_4\pi^- & -v_0\pi^- & -2\Delta_2 \end{bmatrix}. \tag{S8}$$

If we turn on a perpendicular magnetic field generated by the vector potential $\mathbf{A}$, miniaml coupling is achieved by the substitution

$$\pi^- \to \Pi^- = \pi^- - e(A_x - iA_y), \quad \pi^+ \to \Pi^+ = \pi^+ - e(A_x + iA_y). \tag{S9}$$

In the non-relativistic Landau problem, interlevel ladder operators are defined as

$$\hat{\mathsf{a}} = \frac{\ell_B}{\sqrt{2}\hbar}\Pi^- \qquad \hat{\mathsf{a}}^\dagger = \frac{\ell_B}{\sqrt{2}\hbar}\Pi^+, \tag{S10}$$

where $\ell_B = \sqrt{\hbar/(eB)}$ is the magnetic length. For our TLG Hamiltonian $\mathcal{H}_{\mathbf{K}^\pm}$, this amounts to the replacement

$$\gamma_i\Pi^- \to \frac{3\gamma_i a}{\sqrt{2}\ell_B}\mathsf{a}, \quad \gamma_i\Pi^+ \to \frac{3\gamma_i a}{\sqrt{2}\ell_B}\mathsf{a}^\dagger. \tag{S11}$$

The number operator $\hat{\mathsf{a}}^\dagger\hat{\mathsf{a}}$ can be defined as usual, and its eigenstates $|n\rangle$ satisfy

$$\hat{\mathsf{a}}|n\rangle = \sqrt{n}|n-1\rangle, \quad \hat{\mathsf{a}}^\dagger|n\rangle = \sqrt{n+1}|n+1\rangle \tag{S12}$$

The eigenstates of $\mathcal{H}_{\mathbf{K}_\pm}$ can be expressed as

$$\begin{bmatrix} f_{00}|0\rangle + f_{01}|1\rangle + f_{02}|2\rangle + \dots \\ f_{10}|0\rangle + f_{11}|1\rangle + f_{12}|2\rangle + \dots \\ f_{20}|0\rangle + f_{21}|1\rangle + f_{22}|2\rangle + \dots \\ f_{30}|0\rangle + f_{31}|1\rangle + f_{32}|2\rangle + \dots \\ f_{40}|0\rangle + f_{41}|1\rangle + f_{42}|2\rangle + \dots \\ f_{50}|0\rangle + f_{51}|1\rangle + f_{52}|2\rangle + \dots \end{bmatrix}, \tag{S13}$$

where each row of this spinor is an infinite summation and cannot be computed exactly. In our calculation, a proper truncation is imposed such that the summation terminates at a finite n. If we are only interested in the eigenvalues close to zero, a moderate n would be sufficient. For example, the LL diagrams are obtained for $n = 19$. On the other hand, the many-body problem uses $n = 7$.

It should be emphasized that some aspects of the energy spectrum depend sensitively on the system parameters. A precise determination of the parameters is difficult, but a certain degree of consensus has emerged in the past decade. In the absence of Δ_1, the LLs can be labeled as MLG and BLG ones as we have done in Fig. 3f of the main text. More generally, when the MLG and BLG parts are hybridized by nonzero Δ_1, it is more useful to inspect the orbital content of each level. If $|0\rangle$ has the largest weight in Eq. S13, it would be denoted as non-relativistic 0 (NR0). If $|1\rangle$ has a substantial weight in Eq. S13, it would be denoted as non-relativistic 1 (NR1). Naturally, the $\mathbf{K}_\pm, N_{\rm B} = 0$ levels are of the NR0 type and the $\mathbf{K}_\pm, N_{\rm B} = 1$ levels are of the NR1 type. The effects of changing the parameters shall be discussed when we study the many-body problem. The Zeemann coupling is $E_{\rm Z} = g\mu_B B = 0.058g\ B[\text{Tesla}]$ meV with $g = 2$. It is 1.62 meV at $B = 14$ T and is smaller than the energy separation between the NR0 and NR1 levels. Hexagonal boron-nitride has dielectric constants $\varepsilon^{\perp}_{\rm BN} = 3$ in the perpendicular direction and $\varepsilon^{\parallel}_{\rm BN} = 6.6$ within the two-dimensional plane. The interaction between electrons is the screened Coulomb potential

$$V_{\rm SC}(\mathbf{q}) = \frac{e^2}{4\pi\varepsilon_0\varepsilon^{\parallel}_{\rm BN}\ell_B}\frac{2\pi\ell_B}{q}\tanh(qd) = \frac{2\pi\ell_B}{q}\tanh(qd)\,\frac{56.2}{\varepsilon^{\parallel}_{\rm BN}}\,\sqrt{B[\text{Tesla}]}\ \text{meV}, \tag{S14}$$

when d is the distance between the top and bottom graphite gates.

Supplementary Note 2: Methods

In our numerical calculations, electrons are placed on a rectangular torus whose two sides are described by the vectors

$$\mathbf{L}_1 = L_1\widehat{e}_v, \qquad \mathbf{L}_2 = L_2\widehat{e}_y. \tag{S15}$$

The flux magnetic flux N_ϕ through the torus is quantized to satsify $L_1L_2 = 2\pi\ell_B^2N_\phi$. Let us consider non-relativistic electrons with anisotropic mass as described by the single-particle Hamiltonian

$$\mathcal{H}_{\rm NR}(\Lambda) = \frac{1}{2M}\left[\Lambda p_x^2 + \frac{1}{\Lambda}\left(p_y - eBx\right)^2\right]. \tag{S16}$$

The single-particle wave functions are

$$\begin{aligned}\phi^\alpha_m(\mathbf{r}) \;&=\; \frac{1}{\left(2^\alpha\alpha!\sqrt{\pi}\ell_B L_2\sqrt{\Lambda}\right)^{1/2}}\sum_k^{\mathbb{Z}}\exp\left\{-\frac{1}{2\Lambda}\left[\frac{x}{\ell_B}-\frac{2\pi\ell_B}{L_2}\left(m+kN_\phi\right)\right]^2 + i\frac{2\pi y}{L_2}\left(m+kN_\phi\right)\right\} \\ &\times\; H_\alpha\left\{\frac{1}{\sqrt{\Lambda}}\left[\frac{x}{\ell_B}-\frac{2\pi\ell_B}{L_2}\left(m+kN_\phi\right)\right]\right\},\end{aligned} \tag{S17}$$

where $\alpha \in \mathbb{N}$ is the LL index and $m \in [0,\ldots,N_\phi - 1]$. For a generic two-body interaction potential $V(\mathbf{r}_1 - \mathbf{r}_2)$, the many-body Hamiltonian can be written as

$$H_{\rm MB}(\Lambda) = \frac{1}{2L_1L_2}\sum_{\mathbf{q}} V(\mathbf{q}) : \rho(\mathbf{q})\rho(-\mathbf{q}) : \tag{S18}$$

with

$$V(\mathbf{r}_1 - \mathbf{r}_2) = \frac{1}{L_1L_2}\sum_{\mathbf{q}} V(\mathbf{q})\ \exp\left[-i\mathbf{q}\cdot(\mathbf{r}_1-\mathbf{r}_2)\right] \qquad \mathbf{q} = \frac{2\pi}{L_1}q_1\widehat{e}_x + \frac{2\pi}{L_2}q_2\widehat{e}_y \tag{S19}$$

Based on previous experience, it is useful to define

$$|\mathbf{q}(\Lambda)|^2 = \Lambda \left(\frac{2\pi}{L_1} q_1\right)^2 + \frac{1}{\Lambda}\left(\frac{2\pi}{L_2} q_2\right)^2 \tag{S20}$$

and the form factor

$$F_{\alpha\beta}(q_1, q_2, \Lambda) = \frac{1}{\sqrt{\alpha!\beta!2^{\alpha+\beta}}} \sum_{k}^{\min[\alpha,\beta]} 2^k k! \binom{\alpha}{k}\binom{\beta}{k} \left[i\sqrt{\Lambda}\frac{2\pi\ell_B}{L_1} q_1 - \frac{2\pi\ell_B}{\sqrt{\Lambda}L_2} q_2\right]^{\alpha-k} \left[i\sqrt{\Lambda}\frac{2\pi\ell_B}{L_1} q_1 + \frac{2\pi\ell_B}{\sqrt{\Lambda}L_2} q_2\right]^{\beta-k}. \tag{S21}$$

In subsequent calculations, we will need their derivatives

$$\left.\frac{d|\mathbf{q}(\Lambda)|^2}{d\Lambda}\right|_{\Lambda=1} = \left(\frac{2\pi}{L_1} q_1\right)^2 - \left(\frac{2\pi}{L_2} q_2\right)^2 \tag{S22}$$

and

$$\begin{aligned}\left.\frac{dF_{\alpha\beta}(q_1, q_2)}{d\Lambda}\right|_{\Lambda=1} &= \frac{1}{\sqrt{\alpha!\beta!2^{\alpha+\beta}}} \sum_{k}^{\min[\alpha,\beta]} 2^k k! \binom{\alpha}{k}\binom{\beta}{k} \\ \times &\left\{\frac{1}{2}(\alpha-k)\left(i\frac{2\pi\ell_B}{L_1} q_1 - \frac{2\pi\ell_B}{L_2} q_2\right)^{\alpha-k-1} \left(i\frac{2\pi\ell_B}{L_1} q_1 + \frac{2\pi\ell_B}{L_2} q_2\right)^{\beta-k+1}\right. \\ + &\left.\frac{1}{2}(\beta-k)\left(i\frac{2\pi\ell_B}{L_1} q_1 - \frac{2\pi\ell_B}{L_2} q_2\right)^{\alpha-k+1} \left(i\frac{2\pi\ell_B}{L_1} q_1 + \frac{2\pi\ell_B}{L_2} q_2\right)^{\beta-k-1}\right\}\end{aligned} \tag{S23}$$

If $\alpha = \beta$, the second derivative can be simplified to

$$\begin{aligned}\left.\frac{dF_{\alpha\alpha}(q_1, q_2)}{d\Lambda}\right|_{\Lambda=1} &= -\left[\left(\frac{2\pi}{L_1} q_1\right)^2 - \left(\frac{2\pi}{L_2} q_2\right)^2\right] \ell_B^2 \\ \times \frac{1}{\sqrt{\alpha!\alpha!2^{2\alpha}}} \sum_{k}^{\alpha-1} 2^k k! \binom{\alpha}{k}\binom{\alpha}{k} (\alpha-k) &\left[i\frac{2\pi\ell_B}{L_1} q_1 - \frac{2\pi\ell_B}{L_2} q_2\right]^{\alpha-k-1} \left[i\frac{2\pi\ell_B}{L_1} q_1 + \frac{2\pi\ell_B}{L_2} q_2\right]^{\alpha-k-1}.\end{aligned} \tag{S24}$$

To study the FQH states, we keep two LLs with single-particle energy $\epsilon^{(\alpha)}$ and single-particle wave functions

$$|\widetilde{\phi}^{(\alpha)}\rangle = \begin{bmatrix} \sum_{n=0}^{7} f_{0n}^{(\alpha)} |n\rangle \\ \sum_{n=0}^{7} f_{1n}^{(\alpha)} |n\rangle \\ \sum_{n=0}^{7} f_{2n}^{(\alpha)} |n\rangle \\ \sum_{n=0}^{7} f_{3n}^{(\alpha)} |n\rangle \\ \sum_{n=0}^{7} f_{4n}^{(\alpha)} |n\rangle \\ \sum_{n=0}^{7} f_{5n}^{(\alpha)} |n\rangle \end{bmatrix}. \tag{S25}$$

The superscript $\alpha = 0, 1$ labels the two levels, the first subscript $0, 1, \ldots, 5$ labels the components of the spinor, and the second subscript $n = 0, 1, \cdots, 7$ denotes the non-relativistic LL indices. These two levels are called active and other levels are neglected. The creation (annihilation) operator associated with $|\widetilde{\phi}^{(\alpha)}\rangle$ is denoted as $C^\dagger_{\alpha m}$ ($C_{\alpha m}$). The many-body Hamiltonian is

$$\begin{aligned} H_{\rm MB}(\Lambda) &= \sum_{\alpha,m} \epsilon^{(\alpha)} C^\dagger_{\alpha m} C_{\alpha m} + \frac{1}{2L_1L_2} \sum_{\{\alpha_i\}} \sum_{\{m_i\}} \sum_{q_1,q_2} V_{\rm SC}(\mathbf{q}) \exp\left[-\frac{1}{2}|\mathbf{q}(\Lambda)|^2 \ell_B^2 - i\frac{2\pi q_1}{N_\phi}(m_1 - m_4)\right] \\ &\times \widetilde{F}_{\alpha_1\alpha_3}(-q_1, -q_2, \Lambda) \widetilde{F}_{\alpha_2\alpha_4}(q_1, q_2, \Lambda)\, \widetilde{\delta}_{m_1, m_3-q_2} \widetilde{\delta}_{m_2, m_4+q_2}\, C^\dagger_{\alpha_1 m_1} C^\dagger_{\alpha_2 m_2} C_{\alpha_4 m_4} C_{\alpha_3 m_3}, \end{aligned} \tag{S26}$$

where $\widetilde{\delta}$ is a generalized Kroneker function defined as

$$\widetilde{\delta}_{s,t+q_2} = 1 \qquad \text{if and only if} \qquad s \bmod N_\phi = (t + q_2) \bmod N_\phi \tag{S27}$$

and

$$
\begin{aligned}
\widetilde{F}_{\alpha_1\alpha_3}(-q_1,-q_2,\Lambda) &= \sum_{i=0}^{5}\sum_{n_1,n_2}^{7} f_{in_1}^{(\alpha_1)*} f_{in_2}^{(\alpha_3)} F_{n_1n_2}(-q_1,-q_2,\Lambda) \\
\widetilde{F}_{\alpha_2\alpha_4}(q_1,q_2,\Lambda) &= \sum_{i=0}^{5}\sum_{n_1,n_2}^{7} f_{in_1}^{(\alpha_2)*} f_{in_2}^{(\alpha_4)} F_{n_1n_2}(q_1,q_2,\Lambda).
\end{aligned}
\tag{S28}
$$

It is obvious that the total momentum $Y = \sum_i m_i$ is conserved. The low-energy eigenstates of $H_{\rm MB}(1)$ are computed by exact diagonalization. Due to the exponential growth of Hilbert space dimension, the maximal number of electrons is severely constrained. This problem can be partially mitigated if we impose a constraint on the number of electrons in the higher LL. If the total filling factor of the two levels is $3/2$ and there is no interaction, then the lower level is fully occupied and the higher level is half filled. As interaction is turned on, it is possible for all electrons to move to the higher level, but this configuration is not energetically favourable. We shall only retain the configurations in which the higher level does not deviate too much from half filling.

Chiral graviton operators and their spectral functions can be defined as follows [2]. If we choose $\Lambda = 1 + \xi$ and treat ξ as a small parameter, the Hamiltonian can be expanded as

$$
H_{\rm MB}(\Lambda) = H_{\rm MB}(1) + \xi \mathcal{O} \tag{S29}
$$

with graviton operator

$$
\begin{aligned}
\mathcal{O} &= \frac{1}{2L_1L_2} \sum_{\{\alpha_i\}} \sum_{\{m_i\}} \sum_{q_1,q_2} V_{\rm SC}(\mathbf{q}) \exp\left[-\frac{1}{2}|\mathbf{q}(1)|^2 \ell_B^2 - i\frac{2\pi q_1}{N_\phi}(m_1 - m_4)\right] \\
&\times \widetilde{\delta}_{m_1,m_3-q_2} \widetilde{\delta}_{m_2,m_4+q_2} \, C^\dagger_{\alpha_1 m_1} C^\dagger_{\alpha_2 m_2} C_{\alpha_4 m_4} C_{\alpha_3 m_3} \\
&\times \left\{ -\frac{1}{2}\left[\left(\frac{2\pi\ell_B}{L_1}q_1\right)^2 - \left(\frac{2\pi\ell_B}{L_2}q_2\right)^2\right] \widetilde{F}_{\alpha_1\alpha_3}(-q_1,-q_2,1)\widetilde{F}_{\alpha_2\alpha_4}(q_1,q_2,1) \right. \\
&\quad \left. + \left.\frac{d\widetilde{F}_{\alpha_1\alpha_3}(-q_1,-q_2,\Lambda)}{d\Lambda}\right|_{\Lambda=1} \widetilde{F}_{\alpha_2\alpha_4}(q_1,q_2,1) + \widetilde{F}_{\alpha_1\alpha_3}(-q_1,-q_2,1) \left.\frac{d\widetilde{F}_{\alpha_2\alpha_4}(q_1,q_2,\Lambda)}{d\Lambda}\right|_{\Lambda=1} \right\}
\end{aligned}
\tag{S30}
$$

It is more complicated than previously used ones because we have two LLs here [2, 3]. While the physics of gravitons in two LLs may be very interesting, it is more convenient to simplify the problem by going back to one level. If the total filling factor of the two LLs is $3/2$, the eigenstates obtained by exact diagonalization will be projected to the higher level. The summation over α in $\mathcal{O}$ will be limited to the higher level. The operator thus obtained is still not good enough because it cannot reveal the graviton chirality. To this end, we make the substitution

$$
\left[\left(\frac{2\pi}{L_1}q_1\right)^2 - \left(\frac{2\pi}{L_2}q_2\right)^2\right] \quad \Rightarrow \quad \left(\frac{2\pi}{L_1}q_1 \mp \frac{2\pi}{L_2}q_2\right)^2 \tag{S31}
$$

in Eq. (S30) to define chiral graviton operators $\mathcal{O}_\pm$ [these factors appeared in Eqs. (S22) and (S24)]. This replacement is motivated by the connection between chiral graviton operators and anisotropic Haldane pseudopotentials [4].

The eigenstates of $H_{\rm MB}(1)$ are denoted as $|\Psi_n\rangle$ and the associated eigenvalues are E_n. The total weights of the chiral graviton operators are

$$
W_\pm = \langle \Psi_0 | \mathcal{O}^\dagger_\pm \mathcal{O}_\pm | \Psi_0 \rangle \tag{S32}
$$

and the normalized spectral functions are

$$
I_\pm(\omega) = \sum_n \frac{|\langle \Psi_n | \mathcal{O}_\pm | \Psi_0 \rangle|^2}{W_\pm} \delta(\omega - E_n + E_0). \tag{S33}
$$

In finite-size systems with discrete energy levels, $I_{\pm}(\omega)$ can be computed using a method based on Lanczos tridiagonalization and continued fraction [5]. We introduce a rescaled operator $\mathcal{F} = \mathcal{O}_{\pm}/\sqrt{W_{\pm}}$ and define its Green's function

$$G(t) = -i\langle\Psi_0|\exp(iH_{\rm MB}t)\mathcal{F}^\dagger\exp(-iH_{\rm MB}t)\mathcal{F}|\Psi_0\rangle. \tag{S34}$$

A Fourier transform yields

$$G(\omega) = \int dt\;\exp(i\omega t)G(t) = \sum_n \frac{|\langle\Psi_n|\mathcal{F}|\Psi_0\rangle|^2}{\omega - E_n + E_0} = \sum_n \frac{w_n^2}{\omega - p_n}, \tag{S35}$$

where p_n are the poles and w_n^2 are the weights. Using $|F_0\rangle = \mathcal{F}|\Psi_0\rangle$ as the initial vector, the Lanczos algorithm generates a tridiagonal matrix. $E_0 + p_n$ is the n-th eigenvalue of this matrix and w_n is the first element of the n-th eigenstate.

Supplementary Note 3: Results

Let us consider the $\nu = -9/2$ FQH state that occurs without the displacement field. The magnetic field is chosen to be 14 T so we have $\ell_B = 6.842$ nm, $d = 8.697\ell_B$ and $\frac{e^2}{4\pi\varepsilon_0\varepsilon_{\rm BN}^{\parallel}\ell_B} = 31.86$ meV. The LL diagram in Supplementary Figure 8a is computed using the parameters listed above with $\Delta_2 = 0$ meV. It is helpful to begin our analysis with the $\nu = -6$ state. If the interaction is turned off, electrons fill all the LLs below the $\mathbf{K}_-, N_{\rm B} = 0$ level in Supplementary Figure 8a. The presence of interaction is not expected to fundamentally change the $\nu = -6$ state since the single-particle gap below $\mathbf{K}_-, N_{\rm B} = 0$ is about 30 meV. The $\nu = -9/2$ state has an additional 3/2 filling of electrons compared to the $\nu = -6$ state. The most likely configuration is that electrons are spin-valley polarized and fill the $\mathbf{K}_-, N_{\rm B} = 0$ and $\mathbf{K}_-, N_{\rm B} = 1$ levels. It is essential to keep both levels given their separation is only 4.59 meV. The energy spectra obtained by exact diagonalization for the system with 36 electrons and $N_\phi = 24$ are presented in Supplementary Figure 6. Here we only show the low-energy eigenvalues with total momentum $0 \leq Y \leq 11$ because the other half is obtained trivially by magnetic translation. In Supplementary Figure 6a, there are exactly 24 (12) electrons in the $\mathbf{K}_-, N_{\rm B} = 0$ ($\mathbf{K}_-, N_{\rm B} = 1$) level, hence no mixing is present. In contrast, one or two electrons are allowed to escape from the $\mathbf{K}_-, N_{\rm B} = 0$ level in Supplementary Figure 6b,c. For all cases, there are six quasi-degenerate ground states, which is consistent with the prediction for the Moore-Read type states. This information cannot distinguish the Pfaffian, anti-Pfaffian, or particle-hole symmetric Pfaffian states. To this end, we turn to the chiral graviton spectral functions shown in Supplementary Figure 7. For the ground states in Supplementary Figure 6a, particle-hole symmetry in the $\mathbf{K}_-, N_{\rm B} = 1$ level ensures that the two chiralities have the same the total weights. As soon as LL mixing is turned on, W_- becomes much larger than W_+, which suggests that the ground states in Supplementary Figure 6b,c are of the Pfaffian type. In addition, we have also studied the cases with 33 and 39 electrons. While the Pfaffian ground states cannot be defined for them, their chiral graviton spectral functions also reveal that the negative chirality dominates in the presence of LL mixing. On the experimental side, the weak feature observed at $\nu = -5 + 7/13$ can be ascribed to the Levin-Halperin daughter state of the Pfaffian state.

Naively, one may expect to obtain an even-denominator FQH state when a NR0/NR1 doublet has 3/2 filling. This could happen at $\nu = -6 + 3/2, -4 + 3/2, -2 + 3/2, 0 + 3/2$, but only two of them are actually observed. The absence of FQH states at $\nu = -4 + 3/2$ and $-2 + 3/2$ is not easy to explain. It may simply because that the sample quality is still insufficient. Another possibility is the competition between spin-valley polarized states and other states that are not fully polarized. The existence of a nonzero Δ_2 may reduce some single-particle gaps and/or lead to level crossings. We hope that more in-depth experimental and theoretical studies can shed some light on this issue in the future. While the $\nu = 3/2$ state was indeed observed, its nature is more subtle. The active levels are expected to be $\mathbf{K}_+, N_{\rm B} = 0$ and $\mathbf{K}_+, N_{\rm B} = 1$, but the $\mathbf{K}_+, N_{\rm M} = 0$ level is not too far in energy. For our choice of SWMc parameters, the two single-particle gaps between these three levels are 5.22 meV and 14.8 meV. The two gaps can be brought to comparable values by increasing the magnetic field and/or changing the SWMc parameters. The fate of the Pfaffian state in this process is very intriguing.

The $\nu = -3/2$ and $9/2$ FQH states are more difficult to pin down quantitatively. The application of a displacement field results in many LL crossings that is further complicated by a nonzero Δ_2. If the electric field is chosen to be $D = 155$ mV/nm, we would have $\Delta_1 = 17.31$ meV. The evolution of LLs with Δ_2 is shown in Supplementary Figure 8c. For $\Delta_2 = 0$ meV, the active levels at $\nu = -3/2$ and $9/2$ are of the NR0 type. If $\Delta_2 = 0$ meV is fixed but D increases to a very large value or D is fixed but Δ_2 increases to ~ 5 meV, one NR1 level in the $\mathbf{K}_-$ goes up and crosses with one NR0 level in $\mathbf{K}_+$. This enables a Pfaffian type state at $\nu = -3/2$ but the $\nu = 9/2$ state is still mysterious. If Δ_2 is changed to -10 meV, the top most level (in the $-6 \leq \nu \leq 6$ range) would be of the NR1 type. In this case, the $\nu = 9/2$ state corresponds to half filling one spin component of this level, and its mixing with the NR0 type level below it would select the Pfaffian state, as confirmed by the results in Supplementary Figure 9 and Supplementary Figure 10. If we go beyond one-component spin-valley polarized states, there are other even-denominator FQH states. Two examples are the Halperin 331 state [6] and the Jain state constructed from parton theory [7]. In this scenario, there is no need to invoke Δ_2, but our analysis is still applicable for a small nonzero Δ_2. As shown in Supplementary Figure 8b, two NR0 type levels evolve with Δ_1 and become quasi-degenerate in a suitable range. After incorporating the spin degree of freedom, there are four levels whose total filling factor is 5/2. For the integer part 2, electrons could populate the same spin but different valleys or the same valley but different spins. The fractional part 1/2 corresponds to half filling of two NR0 type levels with the same spin or valley index. To realize the Halperin or Jain state, the interlevel interaction should be weaker compared to the intralevel interaction. If the two levels belong to the same valley but have opposite spins, it is difficult to envisage spin-dependent interactions that would realize the Halperin or Jain state. In contrast, the interaction between different valleys may be altered by valley anisotropic terms due to lattice scale corrections to the Coulomb potential [8]. It has been proposed that the Jain state could be realized using suitable valley anisotropic terms [9]. A detailed analysis is required to check if this mechanism also works for the TLG. This is an interesting topic that is left for future works.

Supplementary Figures

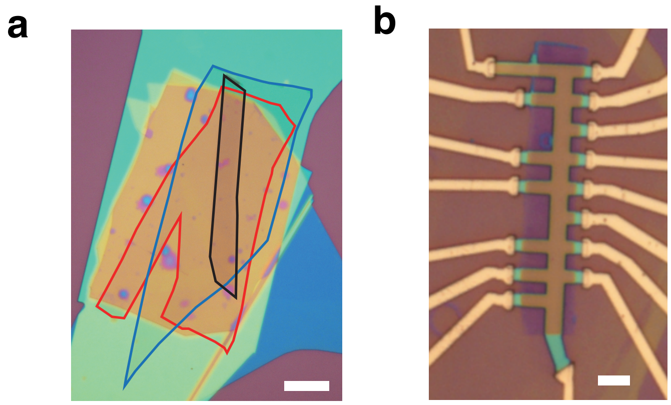

Supplementary Figure 1. **Optical image of one typical device.** **a**, The optical image of the released stack after transferring. The blue (black) line denotes the boundary of top (bottom) graphite and the red line denotes the boundary of trilayer graphene (TLG). The scalebar corresponds to 10 μm. **b**, The completed device with Hall bar geometry that avoids the region with bubbles, after the deposition of Au/Pd/Cr contacts. The scalebar corresponds to 5 μm.

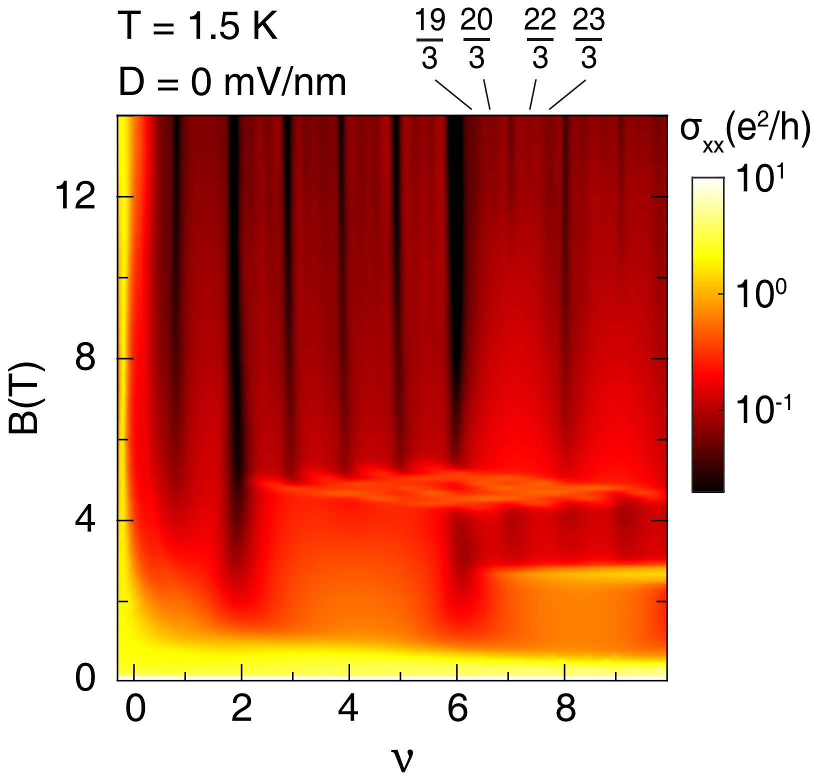

Supplementary Figure 2. **Quantum Hall states in the range 0⩽ν⩽10**. The color map of σ_{xx} measured at at $D = 0$ mV/nm and 1.5 K plotted versus ν and B. The $\nu = 19/3, 23/3$ states appear at a lower B compared to the integer quantum Hall state $\nu = 7$, while the $\nu = 20/3, 22/3$ states appear after it.

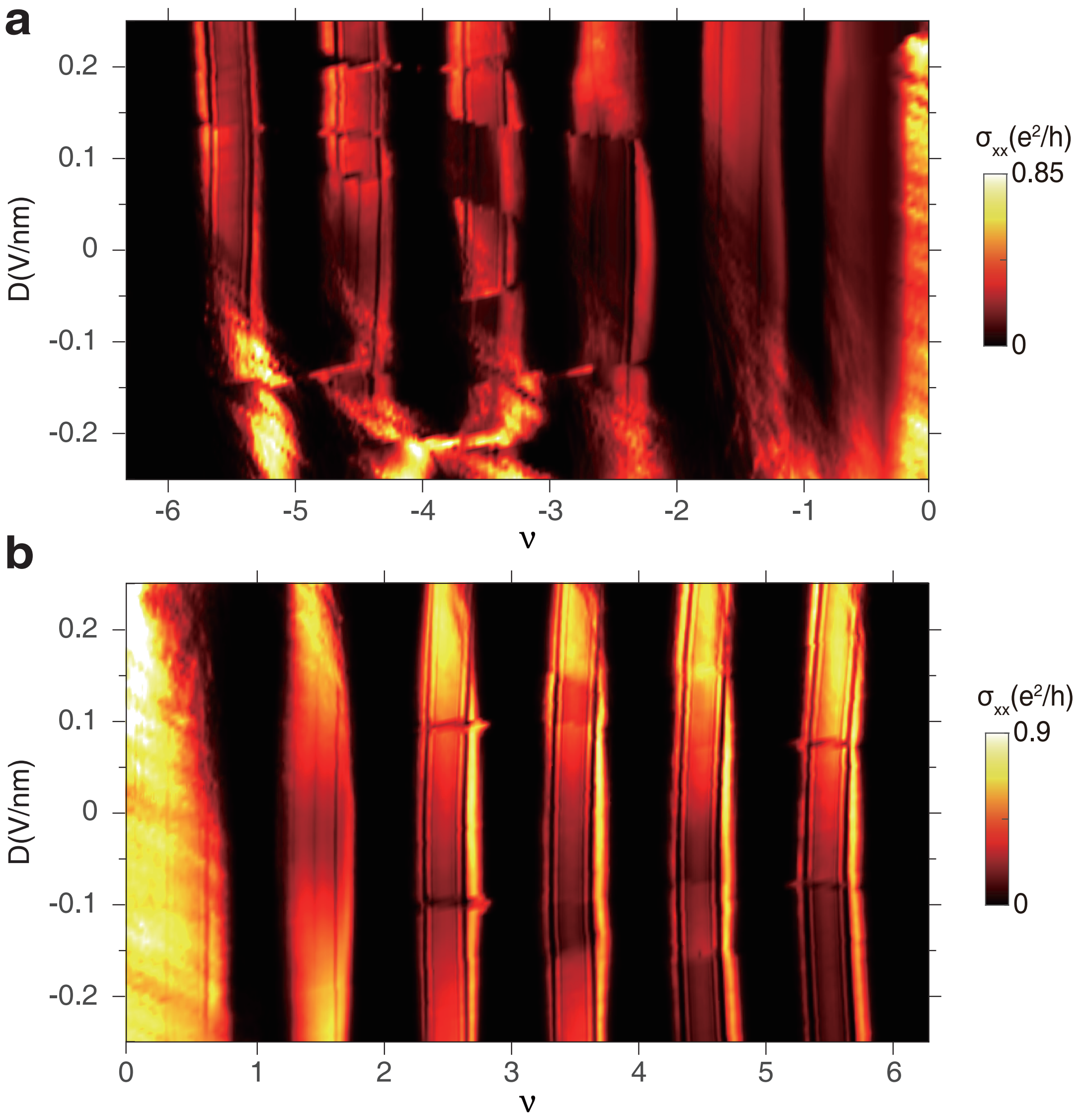

Supplementary Figure 3. **FQH states in the range** $-6 \leqslant \nu \leqslant 6$. **a**, The hole side. **b**, The electron side. Both panels are measured at $B = 14$ T and 15 mK.

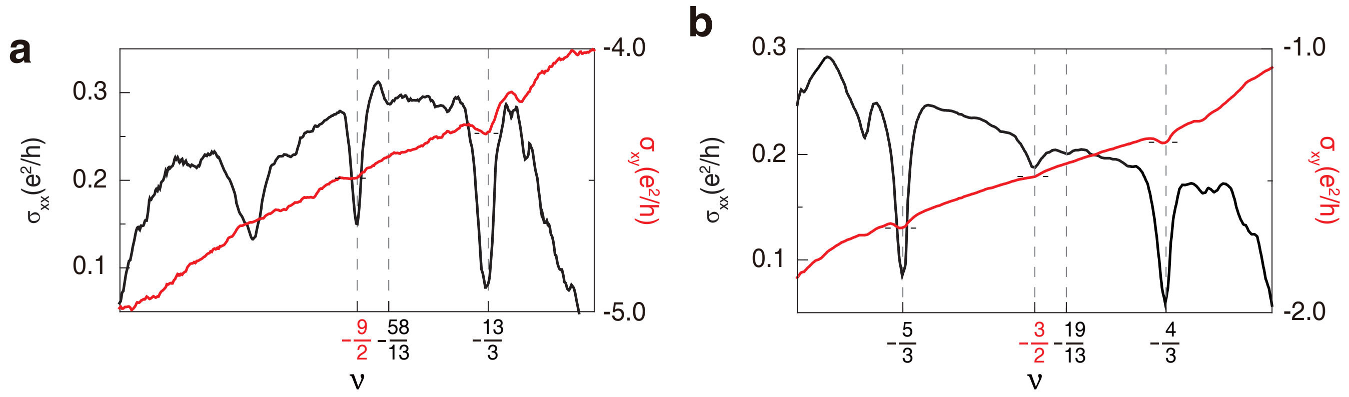

Supplementary Figure 4. **Evidence of the Levin-Halperin daughter states.** **a-b**, σ_{xx} and σ_{xy} versus ν in the range $-5 \leqslant \nu \leqslant -4$ ($B = 14.9$ T and $D = 0$ mV/nm in **a**) and $-2 \leqslant \nu \leqslant -1$ ($B = 14.6$ T and $D = 217$ mV/nm in **b**). Both panels exhibit a small dip in σ_{xx} at $\nu - [\nu] = 7/13$ that may be interpreted as one of the Levin-Halperin daughter states.

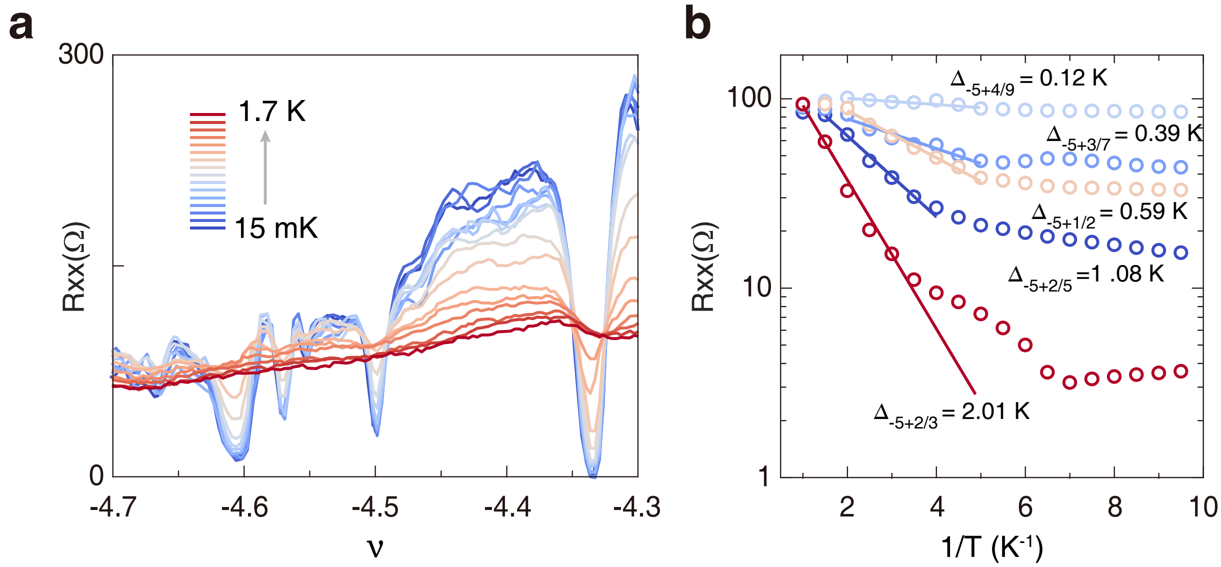

Supplementary Figure 5. **Arrhenius fitting for the gaps of FQH states in the range $-5 \leqslant \nu \leqslant -4$.** **a**, The linecuts of R_{xx} versus ν at different temperatures T. **b**, The Arrhenius fittings for $\nu = -5 + 2/5, 3/7, 4/9, 1/2, 2/3$ using the formula $R_{xx} \sim \exp(-\Delta/2T)$.

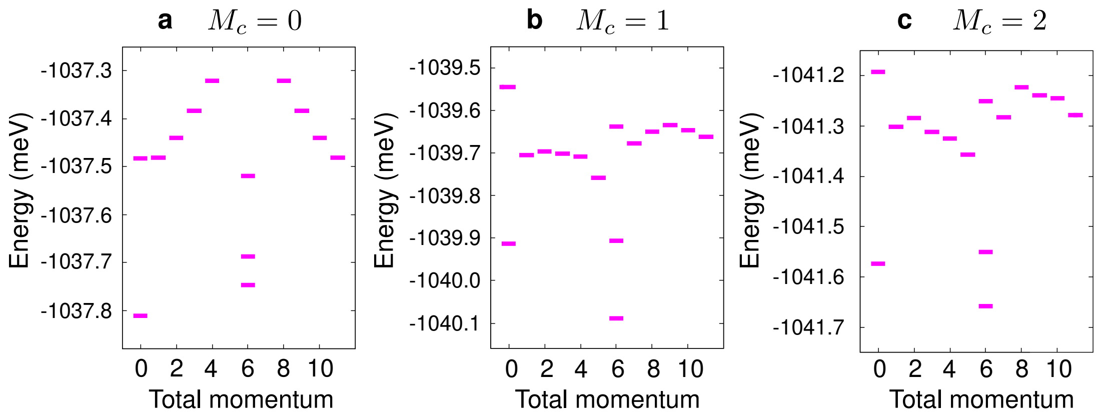

Supplementary Figure 6. **Low-energy eigenvalues of the $\nu = -9/2$ state. a-c,** The eigenvalues for 36 electrons in total with $M_c = 0, 1, 2$, respectively. Here M_c denoted the number of electrons that is allowed to escape from $\mathbf{K}_-, N_\mathrm{B} = 0$.

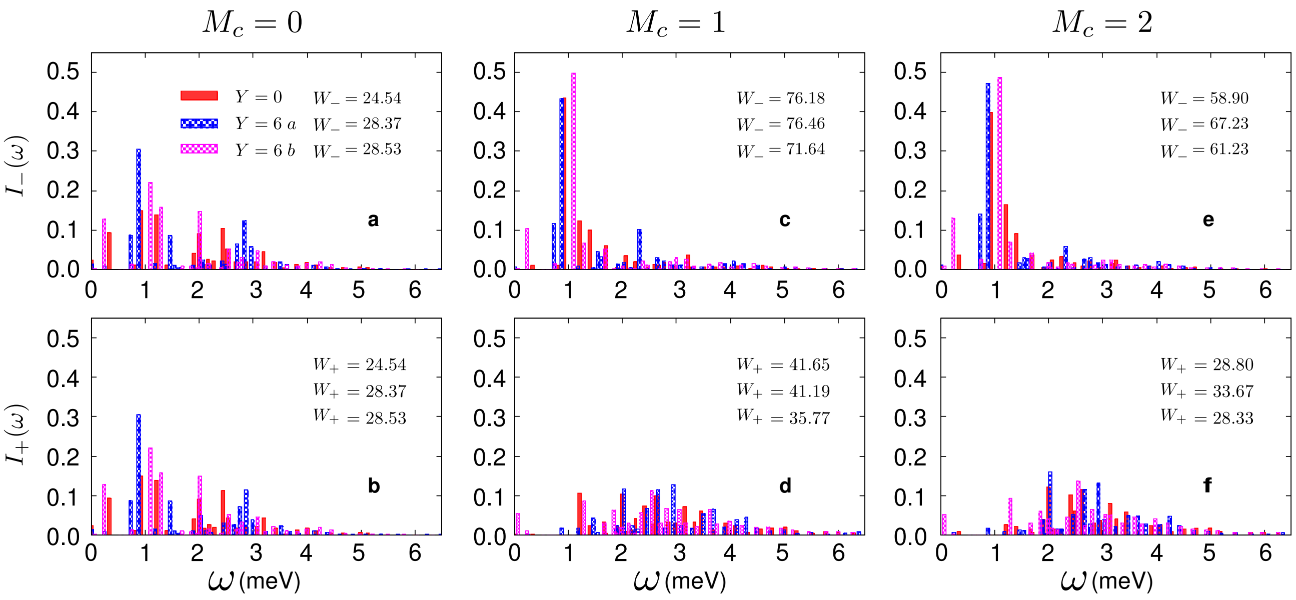

Supplementary Figure 7. **Chiral graviton spectral functions for the $\nu = -9/2$ state. a-f,** The three columns correspond to the states in Supplementary Figure 6. The top row (a, c, e) is for negative chirality and the bottom row (b, d, f) is for positive chirality. In each panel, different symbols are used to represent the three ground states as shown in panel **a**. For total momentum $Y = 6$, the lowest energy state is called a and the second lowest state is called b. The three numbers in each panel are the total weights of the spectral functions.

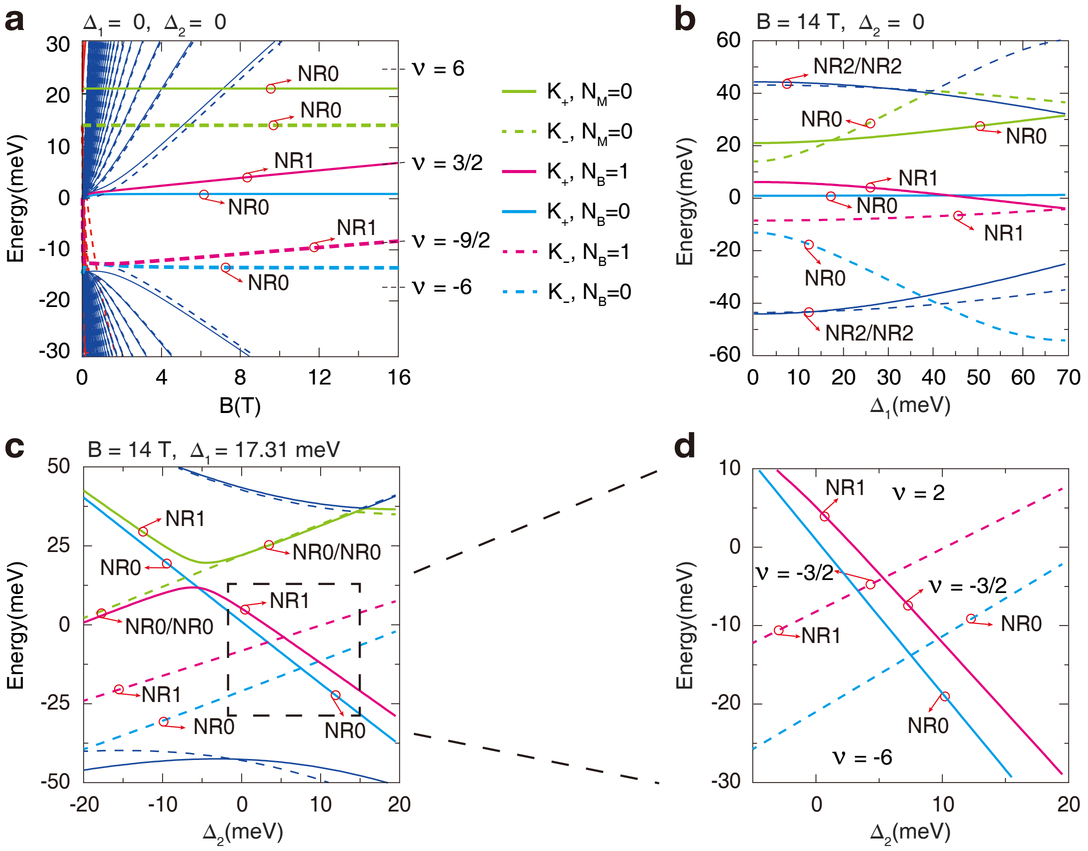

Supplementary Figure 8. **LL diagrams obtained by numerical calculations.** **a**, The same diagram as in Fig. 3f of the main text but plotted in a different way here for comparison. The solid (dashed) lines represent the $\mathbf{K}_+$ ($\mathbf{K}_-$) valley and the spin degree of freedom is neglected for simplicity. The orbital content of a level is indicated as NRx if it has a substantial weight in the non-relativistic LL with index x. **b**, The evolution of LLs with Δ_1 when $B = 14$ T and Δ_2=0. The MLG and BLG levels in panel **a** are hybridized by Δ_1. The green $\mathbf{K}_\pm, N_\mathrm{M} = 0$ levels cross with each other at $\Delta_1 \approx 17$ meV. The blue $\mathbf{K}_+, N_\mathrm{B} = 0$ level and the red $\mathbf{K}_+, N_\mathrm{B} = 1$ level cross with each other at $\Delta_1 \approx 40$ meV. The blue $\mathbf{K}_-, N_\mathrm{B} = 0$ level crosses with two NR2 levels at $\Delta_1 \approx 40$ meV. **c**, The evolution of LLs with Δ_2 when $B = 14$ T and $\Delta_1 = 17.31$ meV. One NR1 level at $\Delta_2 = -10$ meV that may host the $\nu = 9/2$ state is indicated. One NR1 level in the $\mathbf{K}_-$ valley crosses with one NR0 level in the $\mathbf{K}_+$ valley at $\Delta_2 \approx 3.5$ meV. More details about these calculations are given in the Supplementary Information. **d**, Zoom-in view of the region enclosed by dashed black lines in panel **c**. Two possible locations of $\nu = -3/2$ are indicated.

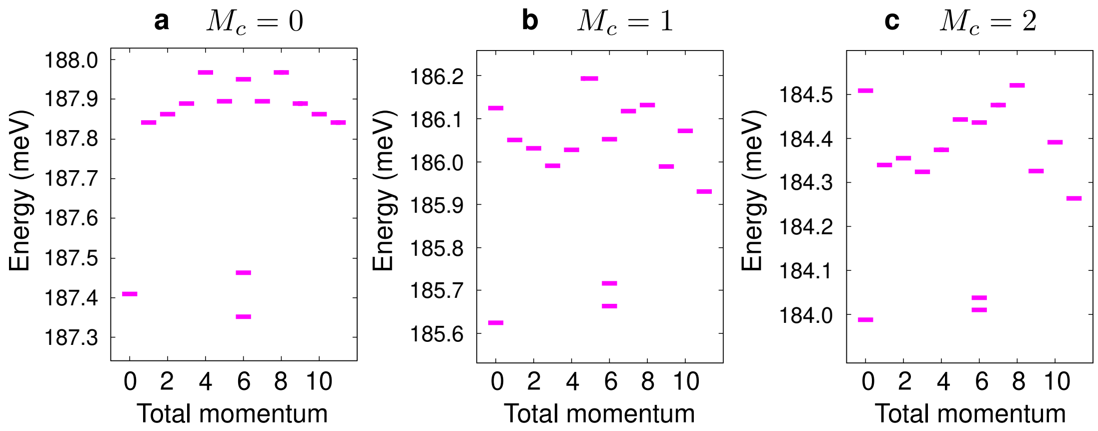

Supplementary Figure 9. **Low-energy eigenvalues of the $\nu = 9/2$ state. a-c,** The eigenvalues for 36 electrons in total with $M_c = 0, 1, 2$, respectively. Here M_c denoted the number of electrons that is allowed to escape from the NR0 level.

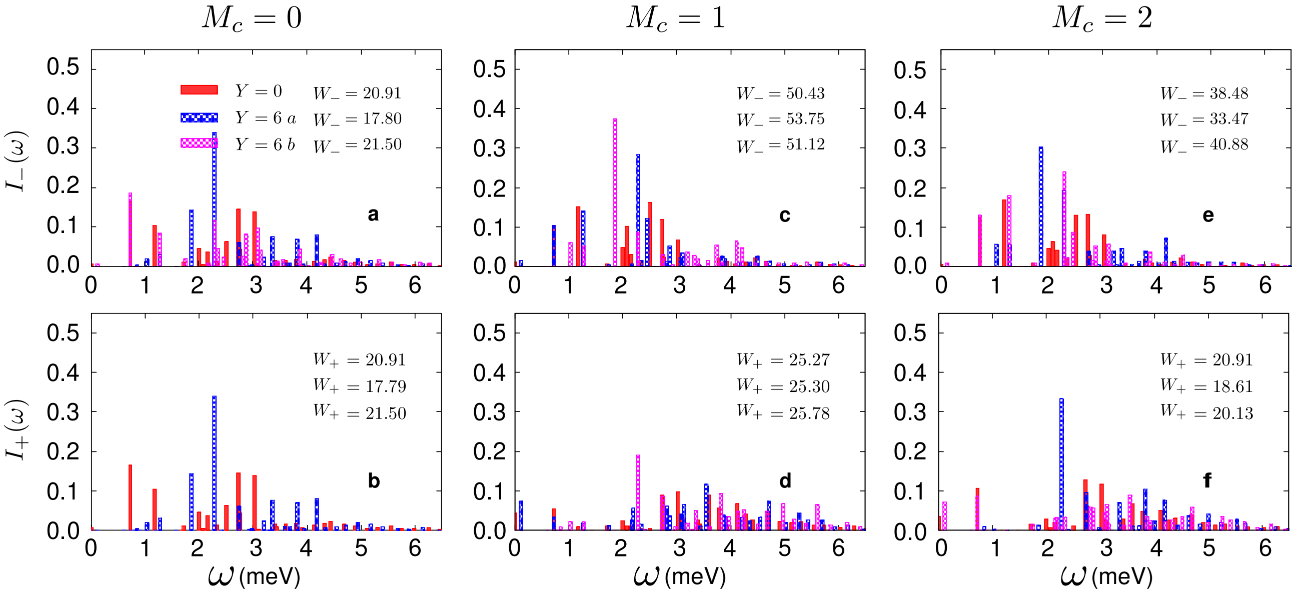

Supplementary Figure 10. **Chiral graviton spectral functions for the $\nu = 9/2$ state. a-f,** The three columns correspond to the states in Supplementary Figure 9. The top row (a, c, e) is for negative chirality and the bottom row (b, d, f) is for positive chirality. The symbols are the same as in Supplementary Figure 7.

Supplementary References

[1] M. Serbyn and D. A. Abanin, New dirac points and multiple landau level crossings in biased trilayer graphene, Phys. Rev. B **87**, 115422 (2013).

[2] S.-F. Liou, F. D. M. Haldane, K. Yang, and E. H. Rezayi, Chiral gravitons in fractional quantum hall liquids, Phys. Rev. Lett. **123**, 146801 (2019).

[3] F. D. M. Haldane, E. H. Rezayi, and K. Yang, Graviton chirality and topological order in the half-filled landau level, Phys. Rev. B **104**, L121106 (2021).

[4] B. Yang, Z.-X. Hu, C. H. Lee, and Z. Papić, Generalized pseudopotentials for the anisotropic fractional quantum hall effect, Phys. Rev. Lett. **118**, 146403 (2017).
[5] E. R. Gagliano and C. A. Balseiro, Dynamical properties of quantum many-body systems at zero temperature, Phys. Rev. Lett. **59**, 2999 (1987).
[6] B. I. Halperin, Theory of the quantized hall conductance, Helv. Phys. Acta **56**, 75 (1983).
[7] J. K. Jain, Incompressible quantum hall states, Phys. Rev. B **40**, 8079 (1989).
[8] M. Kharitonov, Phase diagram for the $\nu = 0$ quantum hall state in monolayer graphene, Phys. Rev. B **85**, 155439 (2012).
[9] Y.-H. Wu, Two-component parton fractional quantum hall state in graphene, Phys. Rev. B **106**, 155132 (2022).
